# Behavior and Body Patterns of the Larger Pacific Striped Octopus

**DOI:** 10.1371/journal.pone.0134152

**Published:** 2015-08-12

**Authors:** Roy L. Caldwell, Richard Ross, Arcadio Rodaniche, Christine L. Huffard

**Affiliations:** 1 Department of Integrative Biology, University of California, Berkeley, Berkeley, California 94720–3140, United States of America; 2 California Academy of Sciences, San Francisco, California, United States of America; 3 Smithsonian Tropical Research Institute, Balboa, Ancon, Panamá, Rep. of Panamá; 4 Monterey Bay Aquarium Research Institute, Moss Landing, California, United States of America; CNRS, FRANCE

## Abstract

Over thirty years ago anecdotal accounts of the undescribed Larger Pacific Striped Octopus suggested behaviors previously unknown for octopuses. Beak-to-beak mating, dens shared by mating pairs, inking during mating and extended spawning were mentioned in publications, and enticed generations of cephalopod biologists. In 2012–2014 we were able to obtain several live specimens of this species, which remains without a formal description. All of the unique behaviors listed above were observed for animals in aquaria and are discussed here. We describe the behavior, body color patterns, and postures of 24 adults maintained in captivity. Chromatophore patterns of hatchlings are also shown.

## Introduction

**Fig 1:**
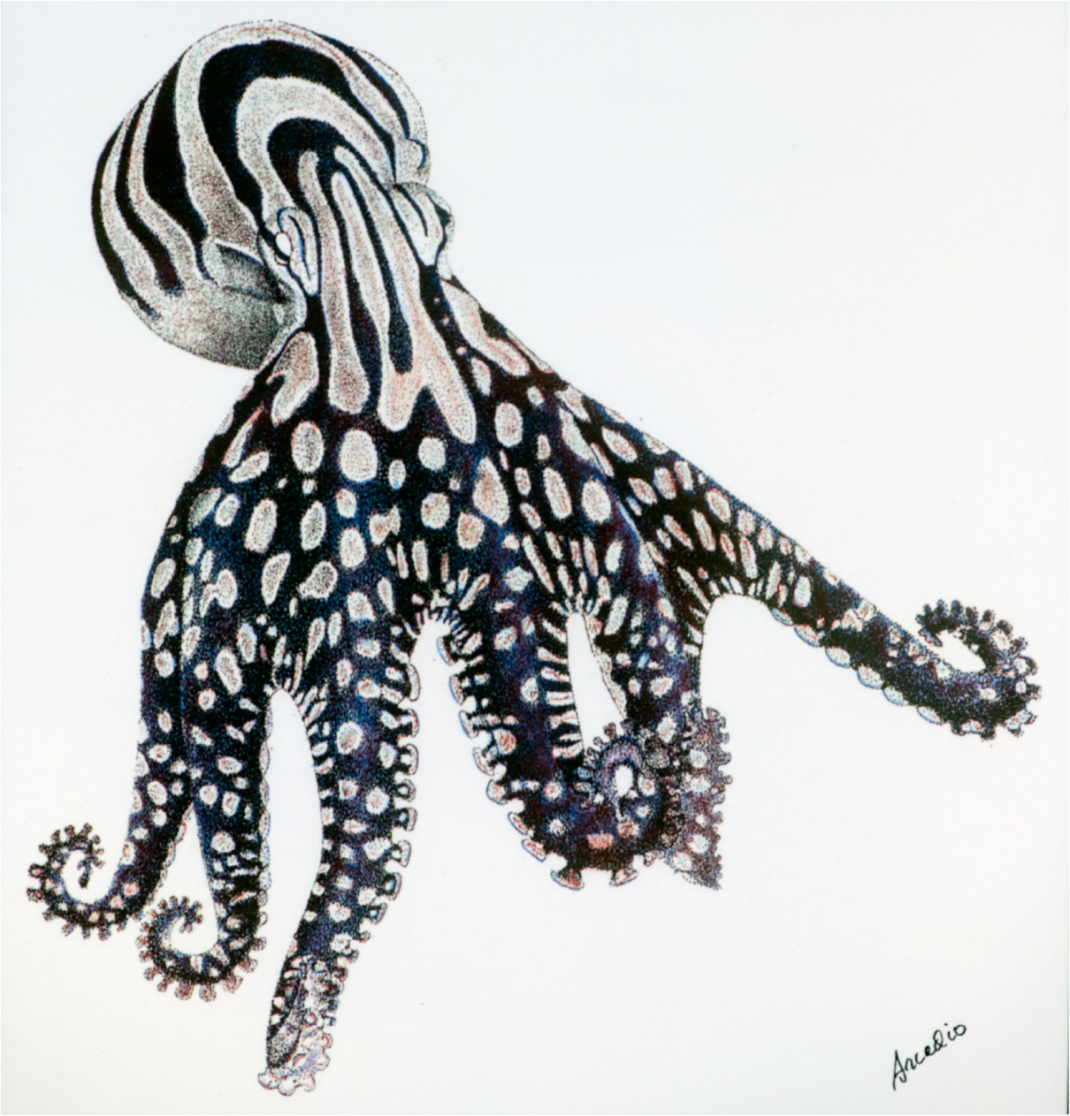
Artwork depicting Larger Pacific Striped Octopus. by AR.

The ‘Larger Pacific Striped Octopus’ (LPSO; Fig 1) first appeared in peer-reviewed literature in 1977, as an illustration of a juvenile without further discussion [1]. Along with *Octopus chierchiae* Jatta, 1889 and *Octopus zonatus* Voss, 1968 from the Western Atlantic and Caribbean [2], LPSO is one of the ‘Harlequin’ octopuses identified by their semi-permanent stripes and spots [3]. The first indications of its unique behavior came in 1982, in a broader discussion of ritualized (body color) patterns in cephalopods [4]. Although mentioned only briefly, early accounts of this octopus based on field collections and groups housed in large tanks listed intriguing behaviors previously unknown for octopuses. These accounts included beak-to-beak mating, dens shared by mating pairs, inking during mating, and extended spawning [4–6]. LPSO was reported to form colonies of 30–40 individuals living at high density, including co-occupancy of dens by pairs presumed to be mates [4,5]. Despite the potential interest of these unusual behaviors to cephalopod biologists, 25 years ago a full ethological description was rejected and the original observations never resubmitted for publication.

In 2012, additional live specimens of LPSO became available through the aquarium trade. Based on observations of these individuals maintained in the laboratory from 2012–2014, we provide photographic and video evidence and behavioral descriptions that confirm many observations reported in previous accounts about this species [4,5], including beak-to-beak mating, co-occupancy of a den by a mating male and female pair, extended spawning, and unique prey-capture methods. This behavioral catalog describes prominent behaviors related to feeding, den construction, mating, aggression, egg-laying, and senescence observable in the laboratory. Body color patterns and postures of adults maintained in captivity, and chromatophore patterns of hatchlings are also shown.

Octopus body color patterns and textures are determined by the presence, location, and pigment composition of fixed anatomical structures and muscles in the skin [7]. While each of these individual components varies in visual intensity from moment to moment, together they ultimately determine the range of body color patterns and postures an octopus can employ, and are thought to offer a species-specific means for identification [8,9]. Although a species description of LPSO is forthcoming by other authors, we provide detailed photographs and descriptions of live individuals that should allow for easy identification, and provide a guide for future ethological studies.

## Materials and Methods

Unless otherwise stated, observations reported here are based on live animals kept in captivity from 2012–2014 at the University of California, Berkeley and California Academy of Sciences, USA. Habitat information from the collector is also provided. We also include limited references to observations from the 1970’s, which are drawn from observations and collections performed by A. Rodaniche at the Naos Island Marine Laboratory of the Smithsonian Tropical Research Institute in the Republic of Panama. At the time, limited observations were made in the field, but most descriptions were based on captive individuals held in the laboratory in March 1975 and May 1977. All observations from the 1970’s are explicitly noted where included or cited.

Between April 2012 and August 2014 a total of 24 individuals (13 males and 11 females) were hand-collected on SCUBA and obtained from commercial aquarium wholesalers [Quality Marine (18), Live Aquaria (1), Russo’s Reef (4) and Sea Logic (1)]. All specimens originated from the same collector from one location in Nicaragua. They were collected for commercial sale in the aquarium trade by Livan, Jansen & Cia. Ltda., Managua, Nicaragua, a firm that holds permits to collect and export octopus. It appeared to the locally-experienced collectors using SCUBA that there was only one persistent aggregation of octopus at the site of collection. Octopuses were shipped to the wholesalers within four days of capture, and then shipped to the authors within four days of arriving in the United States. At delivery they ranged in size from approximately 15–25 mm mantle length (ML). In the laboratory they were initially housed in plastic containers with removable plastic mesh lids (2–4 L). These containers were placed within larger aquaria (227–757 L) with a closed seawater filtration system. This system allowed physical separation of animals in case of aggression because some octopuses can be cannibalistic in captivity [10] and in the wild [11,12]. This housing system also maintained the possibility for visual and chemical communication between individuals. Water temperature was 20–23°C. Octopuses were fed every other day a variety of crustaceans and molluscs (see Hunting and feeding section). Because animals were active in the day, and did not appear to be active during the night (as verified by haphazardly timed checks), ambient light was sufficient for observations.

After several successful matings in controlled environments that resulted in no cannibalism or damage beyond sucker marks on the mantles of males, four male and female pairs were allowed to share a tank. In each of these cases the female was initially kept in her den in a plastic container with the top removed, and the male was allowed access to the entire aquarium. After several weeks of that arrangement, the female’s den was removed from the container and placed in the main tank space. After the last shipment of LPSO was received, a female/female/male trio was housed together. A female/female paring was attempted but was quickly abandoned when the larger female started to eat the eggs she had already laid. A male/male pairing was never attempted. These groups were housed in tanks ranging from 113–454 L with sand substrates and small objects for shelter (see Distribution, habitat and denning information below).

Photographs were taken of animals in aquaria with a variety of camera gear. Still images were taken with Nikon D7000, Nikon D800, AF-S MicroNIKKOR 105mm, and Nikon AF-S DX NIKKOR 18–200mm with a Nikon SB 600 strobe (RR), and a Nikon D800 with Nikon 910 strobes (RLC). Videos were taken with a Sony HDR-CX165 (RR), a Sony Handycam HDR-XR260-V (RLC) and an iPhone 4 (RR).

We used conventional terminology of gross morphology [13]. Terms used to describe skin morphology follow those defined and illustrated previously [8,9,14–21]. Behavioral terminology follows descriptions in the literature for other species, or were defined here if no established term was available. Modes of locomotion follow those outlined previously [22]. Because we aimed to observe as many behaviors as possible, we were not able to control for observation effort of each animal. Therefore any references to the frequency of observations must be interpreted with caution.

This non-invasive study was carried out in accordance with the policies of care outlined by the Association of Zoos and Aquariums, and the Steinhart Aquarium’s Animal Research Committee and Animal Care Committee under the guidance of the Steinhart Aquarium’s Animal Health Department. This study did not require approval of protocols by The University of California, Berkeley Animal Care and Use Committee (our IACUC), which only requires approved protocols for vertebrate animals (Animal Care and Use Program Policy http://www.acuc.berkeley.edu/policies/acu_program.pdf). At this time, and during the period that the research was conducted, no Federal, State or Local regulations have been applied to the study of cephalopod mollusks. We therefore did not seek institutional approval. All animals were provided with an appropriate den in which to reside, and fed live shrimp, crabs, snails and clams. No animals were euthanized. They all died a natural senescent death typical of octopus after reproducing. When males and females were introduced to observe mating and other reproductive behavior, the animals were closely monitored and at any sign of aggression the animals were immediately separated. No damaging injures occurred.

Animals were offered food the day after arrival, but were allowed to acclimate to new conditions for several days to a week before a mating opportunity was presented. Because of the close proximity of tanks, many of the octopuses could see each other during this acclimation period, but were not able to physically interact.

## Results

### Distribution, habitat, and denning

LPSO appears to be endemic to the tropical Eastern Pacific. In the 1970’s this species was common in the Bay of Panama, Panama, and commercial shrimp trawlers found individuals on the northern Pacific coast of Columbia. Additional individuals have been collected in Guatemala (Smithsonian Institution Invertebrate Zoology USNM 817793, a single individual incorrectly identified as *Octopus spilotus*) and Magdelena Bay, Baja Sur, Mexico (Dr. Gustavo Hinojosa Arango, personal communication). LPSO occupies regions with a soft mud substrate, or a mix of mud and sand. Trawls capturing individuals in the 1970’s operated at depths ranging from 7–100 m, however exact depths of capture during those collections are not known. Based on earlier collections, the deepest published mention of LPSO habitat is 300 m [4], and groups of up to 40 individuals were observed living in close proximity, with dens within one meter of one another [4]. The aggregation found in Nicaragua in 2012 appears to have persisted for longer than two years. Large-scale population density and sex ratios are not known.

In the laboratory, individuals occupied dens of a variety of natural (shells of *Nautilus*, *Strombus*, and the barnacle *Conchylepes*) and artificial (terra cotta flower pots, glass bottles, custom blown glass, PVC tubes 3, 3.8 and 5 cm diameter) materials. Although available, pebbles were generally not used for construction of a larger den. The only individual to use them for denning was a senescing female that hid behind pebbles in the corner of a tank for the four weeks prior to her death. This female was observed to build a small rounded mound of sand around the den entrance, sometimes burying the entrance to the den (15 to 31 cm in diameter, up to 5 cm deep in the center). In cases when air bubbles were trapped in barnacles and conch shells used for dens (as occurred sometimes during tank cleaning), octopuses turned the shells over, which removed the air bubbles, before entering. Sand that entered the den was removed from the den with forceful jetting of water through the funnel. After feeding, the remains of prey items were forcefully ejected from the immediate vicinity of the den entrance (S1 Movie). Octopuses frequently moved 1–2 body lengths before ejecting prey remains. This observation supports observations from the 1970’s that conspicuous prey remains were not found near dens in the wild.

Males were observed to move into different dens at irregular intervals, and would sometimes forgo a den all together, roaming the aquarium or settling in the upper corners of the tank. Females typically occupied the same den for the entire duration of captivity, especially after beginning to lay eggs. In one case, a female occupied the same den for over 14 months, and in cases where females were moved to different tanks, their den was moved with them.

One male and female pair housed together (of the four male-female pairs that shared a tank) generally spent most of their time in separate dens, but occasionally both animals occupied the same den for several hours at a time. In these cases the male foraged and explored away from the shared den for several hours, sometimes returning to it, sometimes not. On at least four occasions, this pair shared a den for more than three consecutive days. These dens were PVC tubes with more than one opening. When this male and female shared a den, each one sat at a different opening, and the pair mated daily. This pair was also observed to share food (see Hunting and feeding section below). Multiple occupancy of a den was not observed for the Female/Female/Male group sharing a tank.

### General body patterns and postures

**Fig 2:**
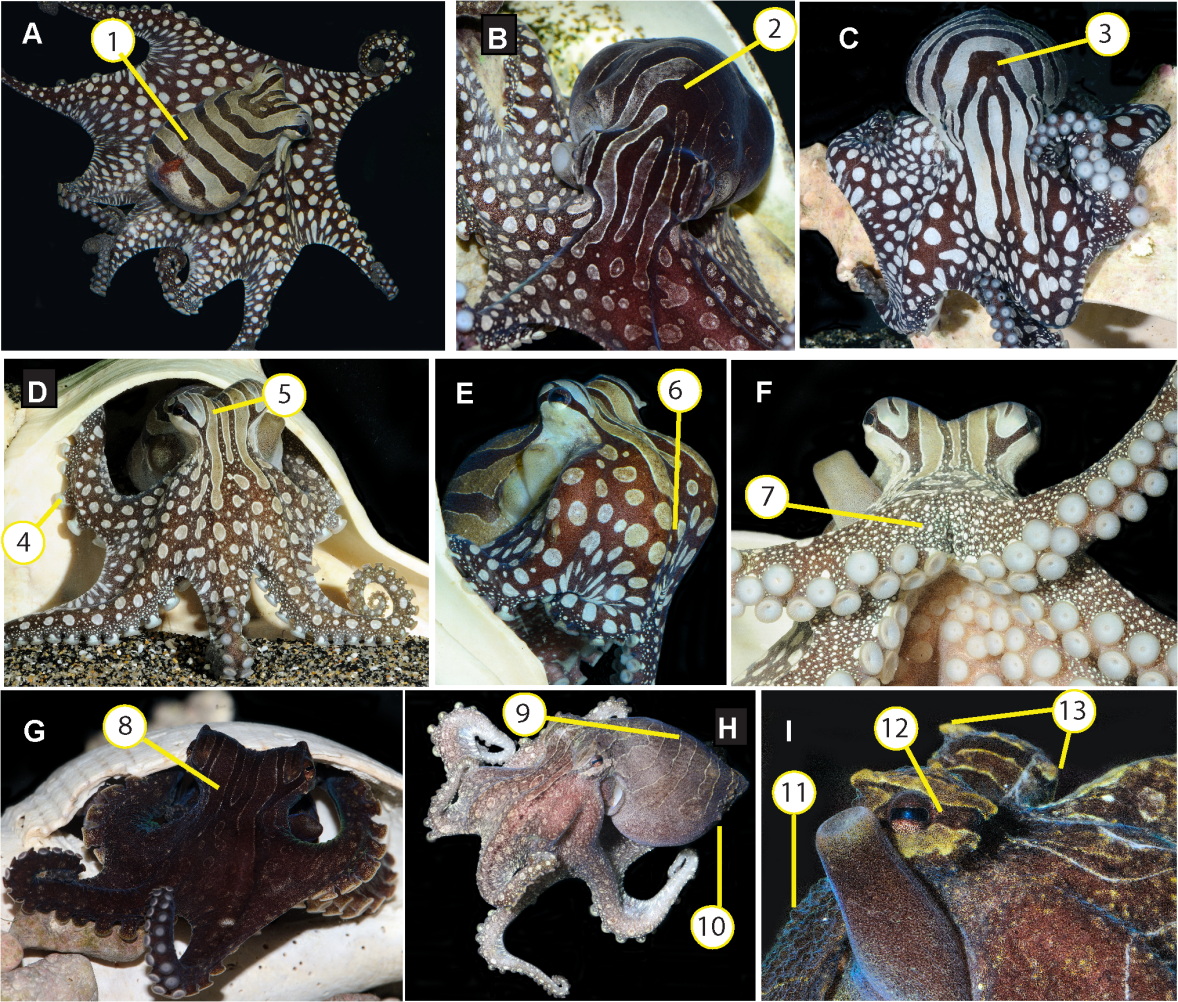
Body color and texture components and patterns of the Larger Pacific Striped Octopus. A) *Spot-and-striped* display with flared body posture, showing longitudinal head stripes and (1) dark horizontal mantle bars; B) Bilateral display with dark body pattern on the left of the individual’s midline (viewer’s right) (2), and *spot-and-stripe* body pattern on the right of the individual’s midline (viewer’s left); C) *Spot-and-stripe* display highlighting (3) region with individual variation in body color components; D) Dorsal arm crown and arms in *spot-and-striped* body color pattern showing (4) white sucker rims and (5) pale longitudinal stripes on head, with raised pale border; E) Lateral view showing smooth skin and (6) large white spots on web, arm crown and arms; F) White spots on the raised bumps of granular skin texture, on dorso-lateral edges of arms (7); G) Dorsal arm crown and arms in dark color pattern showing (8) dark stripes with pale raised border; H) Dorso-lateral view in pale color pattern showing (9) raised pale border between horizontal mantle bars on dorsal mantle and (10) small mantle papillae; I) Eyes and funnel showing (11) granular skin texture, (12) dark eye bar, and (13) supra-(left) and sub-(right) ocular papillae. Note blue sheen of funnel and green undertone of pale bars on the eye. All photos by RLC.

LPSO in the laboratory expressed a moderate number of skin components and textures. Body color patterns were primarily represented by a gradient between *all pale*, *all dark brown*, and *stripe-bar-spot*. The *stripe-bar-spot* pattern was dominated by full coverage of alternating brown and white bars and stripes on the head and mantle, and white spots over a dark brown background on the arm crown and arms. Dark-light contrast of these components was expressed in varying degrees from high contrast (Fig 2A–2E) to barely visible during expression of *all dark brown* (Fig 2G) and *all pale* (Fig 2H). When in adjacent tanks, individual LPSO frequently expressed the high-contrast *stripe-bar-spot* while the neighbor was feeding or otherwise visibly active in its tank. *Stripe-bar-spot* could vary bilaterally (Fig 2B; S2 Movie). The pale bars and stripes of this pattern were delineated by a raised white border (1 mm maximum in elevation) that circumscribed the pale components. These bars and stripes varied in shape between individuals, especially on the head and anterior portion of the dorsal mantle. Large pale spots over a uniform brown background covered the aboral surface of the web and arms (Fig 2A–2E). Smaller white spots corresponding to the raised portions of granular skin texture were visible on the dorso-lateral surfaces of the arms (Fig 2F). A blue sheen was often visible on dark areas (Fig 2I), and a green undertone visible in the pale regions. A dark eye bar (Fig 2I) was visible in all body color patterns, and sometimes extended anteriorly and/or posteriorly beyond the eye. The hectocotylus groove was visible as an unpigmented line along the dorsolateral edge of the third right arm. Skin texture was granular with the following papillae: posterior mantle papilla, ventral mantle papillae, supra-ocular papillae and sub-ocular papillae. Papillae shape was conical rather than flap-like or branched.

**Fig 3:**
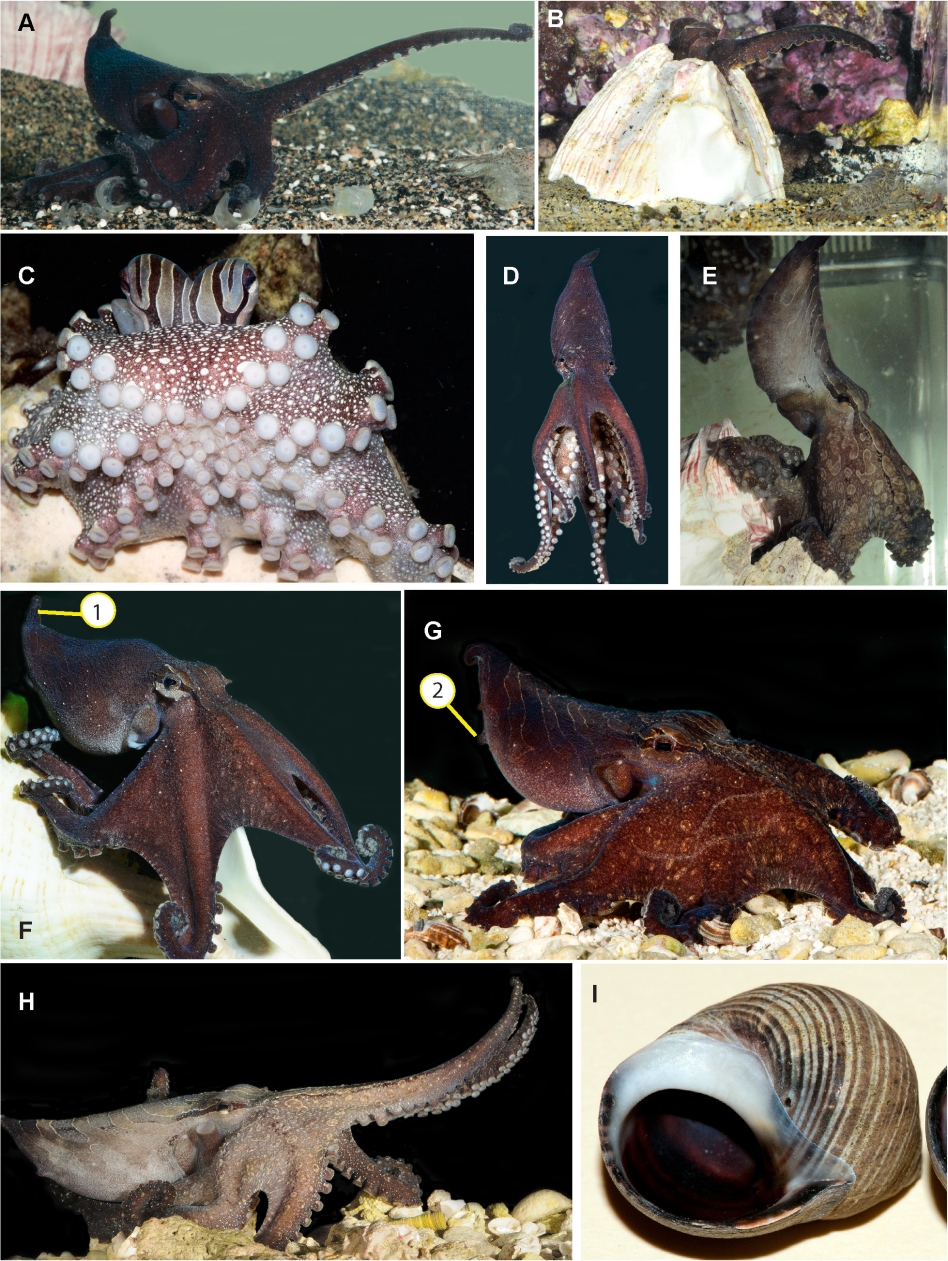
Body postures of the Larger Pacific Striped Octopus. A-B) dorsal arm reach while hunting in the open (A) and from a shelter (B); C) Sitting with oral surface of web exposed and arms held behind mantle; D) Upright swimming; E) Upright crawling with reflexed mantle; F) Upright crawling with reflexed mantle, domed arm crown, and (1) posterior mantle papilla; G) Forward crawling with reflexed mantle, flared arms, and (2) ventral mantle papillae; H) *Slow bounce* with pale color pattern, extended eye bar, erect dorsal arms (arm pair I) reflexed at tips; I) position of drill hole in snail shell. All photos by RLC.

LPSO expressed a diversity of body postures in the laboratory (Fig 3). At rest, individuals often sat with the oral surface exposed and the arms pulled back behind the mantle (Fig 3C). Brooding females also assumed this posture most of the time they were with their eggs. In this posture, brooding females tended their eggs with roving arm tips. In the presence of females, either before or during mating, and when feeding, males frequently moved the distal portion of the arm tips in an irregular twirling motion (S2 Movie, S3 Movie). The mantle was extended, arched, and drawn to a point in many body postures during hunting, swimming (Fig 3D), and crawling (Fig 3E–3H). At times, while crawling, the dorsal arms (arms I) were held forward with the arm tips curled back tightly exposing the suckers (Fig 3E). In a posture we call *slow bounce*, in which animals exhibited darker coloration, flattened body with the dorsal arms were held forward, while the upwardly-arched arms and mantle were alternately raised and lowered slowly (Fig 3H). This posture was commonly expressed by animals away from their dens, primarily in the early morning or when tank water was cloudy due to aquarium maintenance. *Slow bounce* was often associated with slow movements forward, incorporating an erratic bouncing motion, and a flared arm crown (S4 Movie). When hunting shrimp, a dorsal arm (always arm LI or RI) was extended in an arched fashion and curled at the tip to expose the suckers outward (see Hunting and feeding below; Fig 3A and 3B; S5 Movie).

Numerous skin components common among shallow-water octopuses and diagnostic of taxonomic placement [8,23–25] were not observed in LPSO: dorsal mantle white spots, frontal white spots, a white “V” of leucophores at the posterior end of the dorsal mantle, longitudinal stripes along the dorso-lateral edge of the arms, dark arm bars, ocelli, neck dark spots, star-like pattern around the eye, primary papillae in a diamond configuration, flaplike primary papillae, branched papillae, or pronounced patch-and-groove skin texture. *Passing cloud* [23] was not observed.

### Hunting and feeding

In the 1970’s individual LPSO were offered a range of locally available crustaceans to consume *ad libidum*. At the time, shrimps (*Peneus vannemai* and *P*. *occidentalis*) were taken before stomatopods (*Lysiosquilla panamica* and *Squilla aculeata*), which were taken before crabs (*Callinectes arquatus* and *C*. *toxotes*). Bivalves (*Venerupis philippinarum*) were pulled apart or crushed when small, while a larger bivalve was drilled, as with *Octopus dierythraeus* [26]. Individuals observed in 2012–2014 consistently drilled *Littorina* shells near the operculum (Fig 3I). The location of each drill site on the snail shell was consistent for each individual LPSO but varied between individuals. Scanning electron micrographs of two drill holes by a single individual showed a primary beveled hole with a triangular notch on one side, with an outer diameter of 1/3 mm and an inner diameter of 200 microns.

LPSO in the laboratory appeared to be visual predators stalking and/or chasing down live prey. Shrimp were caught by extending a dorsal arm slowly, in an arched fashion, lowering it over the front end of the shrimp and touching it on the carapace. During this behavior the hunting arm was curled back at the tip so that the suckers were facing outward and grabbed the shrimp, typically as it retreated backward toward the octopus when tapped (S5 Movie). Arm extension was often associated with slow approach toward the shrimp, with LPSO only making contact with the prey when the tip of the arm had passed over the shrimp. Crabs were not pursued using this single arm extension behavior. Instead, crabs were captured when LPSO pounced on them directly.

**Fig 4:**
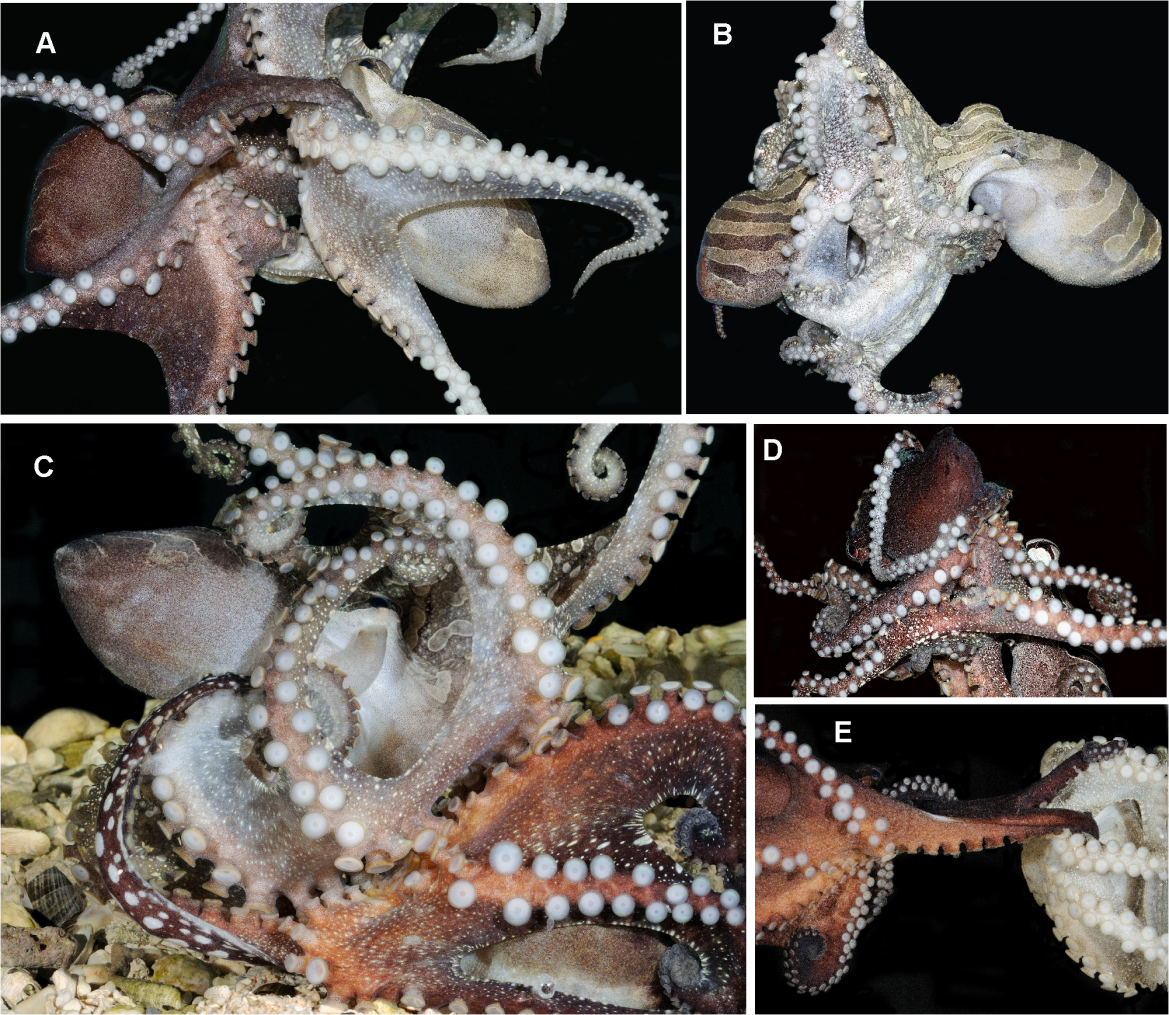
Beak-to-beak mating in Larger Pacific Striped Octopus. A) Insertion of male hectocotylized arm into female mantle cavity, male on left, female on right; B) Female (right, pale) slightly enveloping male (left) during mating; C) Sucker alignment during mating; D) sucker alignment and mantle encircled with arm during mating; E) Hectocotylus insertion during ‘distance’ mating in beak-to-beak posture (male left, female right). All photos by RLC.

The one male-female pair that shared a den was regularly observed to feed in the beak-to-beak posture. In these cases the female initially caught the prey item and the male approached, entering into the beak-to-beak position, with the prey between the two animals. This pose was held for 5–10 minutes, during which several of the arms of each animal would line up sucker to sucker (as with the non-feeding individuals in Fig 4C). It appeared that both animals were eating while in this position. Copulation was never seen during these feeding events, but occurred at other times. Because we did not want to sacrifice individuals, we did not perform gut content analysis, which would have been required to confirm feeding by both individuals.

### Mating behavior and agonistic interactions

Mating almost invariably occurred beak-to-beak and involved agonistic behavior (Fig 4; S6 Movie). During the first case of mating observed, animals were separated. In all other cases the male-female pair completed mating undisturbed. During mating, females consistently exhibited *all pale* body patterns, and males consistently exhibited a darker or higher-contrast display. Mating typically began with the male approaching the female and inserting the hectocotylus into the female’s mantle, in a beak-to-beak position. In some cases the male and female sat briefly facing each other with the suckers aligned (Fig 4C and 4D). When observed, this behavior did not last more than a few minutes, and typically preceded mating. During mating, females often enveloped the male either partially (Fig 4B) or fully into the web, with the male’s arms bent backward over his mantle. Females were typically larger than the males offered. Mating individuals also frequently grappled arms, females jetted water at males through the funnel, and females pushed males away. The most overt example of female aggression during mating took place when a male attempted to mate with a brooding female. However, other brooding females mated without overt resistance. When brooding females mated, the beak-to-beak position permitted the female to remain in the brooding area with the oral surface facing the male and her mantle facing the eggs. Following release of males from mating, sucker marks from the grip of the female were often visible on his mantle. While the hectocotylus was inserted, spermatophores were often seen being flushed from the female’s mantle cavity or funnel. It was not possible to determine whether these spermatophores had been flushed from the female’s oviducts by forceful female mantle contractions, or if the spermatophores had never been placed into the oviduct by the male in the first place. In one of the two cases of inking observed of all animals in captivity, a male inked when enveloped by the mating female. *Arm pulling* [27], *constricting* [28], and cannibalism were not observed. Mating pairs were not observed to swim or crawl while mating.

Males were not housed together, and as such we did not have the opportunity to observe- possible male-male aggression. No direct aggression was observed of females housed in the female/female/male group tank.

### Locomotion

Primary modes of locomotion included crawling, jetting, and swimming. Crawling took place using a variety of body postures and patterns, including the *slow bounce* posture which included bouncing while crawling (S4 Movie). Jetting individuals were uniformly pale or dark brown, and were not observed to exhibit complex body patterns. LPSO appeared to exhibit head-first swimming primarily when approaching prey, or when males approached a female to mate. *Dorso-ventrally compressed swimming* and *bipedal walking* [29] were not observed.

### Egg laying and brooding

**Fig 5:**
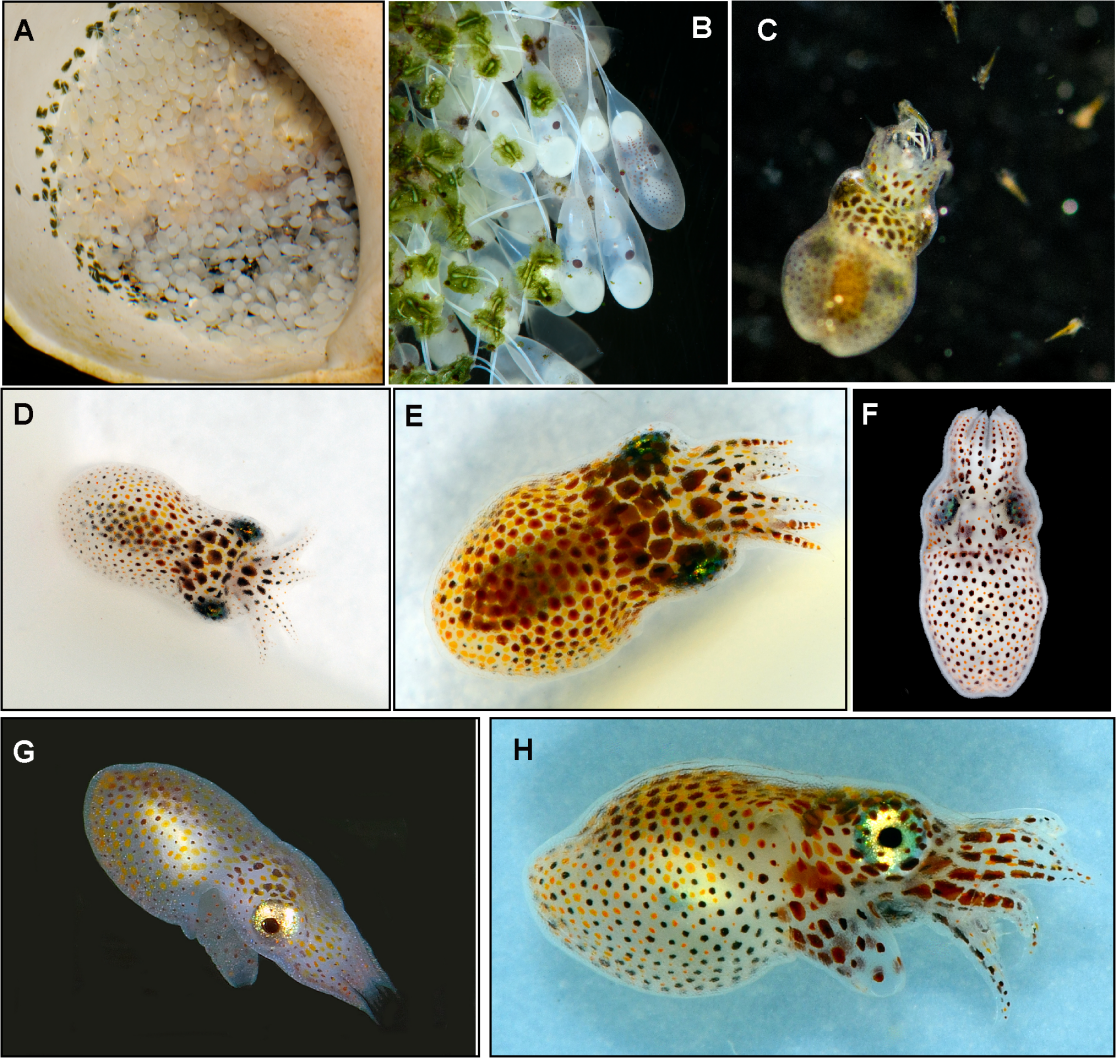
Life cycle stages of Larger Pacific Striped Octopus. A) Eggs attached to shell used for brooding; B) Close up of eggs from a single clutch. Note embryos at different stages of development and eggs attached in pairs; C) two day old hatchling eating 1–4 day old *Lysmata amboinensis* larvae; D-F) Hatchlings one day old—D) dorsal view; E) oblique lateral view; F) ventral view; G-H) two-day old hatchling after feeding on stomatopod larvae—G) dorsal view; H) lateral view. A, B, D, E, G, H by RLC; C, F by RR.

In the 1970’s, three females were captured by trawl with what might have been their eggs on the inside of gastropod shells (1—*Ficus ventricosa*, 2—*Malea ringens*). In 2012–2014 regardless of den type used in captivity, females moved into dens and laid eggs inside. All eggs laid in captivity (n = 5 females) were fertile, even those spawned over four months after the last mating. Eggs appeared to be laid two at a time, and affixed in pairs to the wall of the den by a stalk tipped with adhesive (Fig 5A and 5B). Once a female began laying eggs, spawning extended through to the beginning of senescence. Once hatching began it continued daily for over three months (n = 5 females), suggesting eggs were also laid daily. One female spawned continuously for six months and brooded for a total of eight months. Spawning in LPSO has been referred to as iteroparous [5] because females continue to mate and feed during this very extended spawning period. However, LPSO appear better designated as ‘continuous spawning’ with a single prolonged egg-laying period, rather than ‘iteroparous’ with multiple discretely separate egg-laying periods [30]. One female was observed to have eggs in both a den and on the walls of the plastic container and would carry the den with her, over her mantle, to tend both sets of eggs. When brooding females were found away from their eggs, they usually immediately returned to their eggs and maintained arm contact with them until we left. Females frequently tended eggs by running arm tips through them to presumably keep them from fouling.

Two females were introduced to males while brooding. One of these females accepted all eight separate mating opportunities. The second brooding female accepted matings on three occasions, but rejected attempts by two different males. These rejections by the second female might have taken place during early senescence.

### Growth, senescence and life-span

Growth is poorly known in LPSO. A single individual obtained at 60 mm ML in the 1970’s grew to 110 mm ML in 282 days. Maximum size attained in captivity in 2012–2014 was 45 mm ML for males and 70 mm ML for females. Senescence, marked in octopuses by the abrupt cessation or gradual reduction in feeding [31], appeared to take longer for females than males. Males typically died one to two weeks after they abruptly ceased feeding (though three weeks in one case). Females typically died between two and four months after showing the initial signs of senescence. At the onset of senescence, females gradually reduced food intake and hunting behavior, eating smaller amounts of prey, less-frequently, and gradually reduced general activity. Females that began senescence while caring for eggs never stopped caring for the eggs throughout senescence. Impaired color change was gradual in females, first noticeable two weeks into senescence. Three to four weeks into senescence, resting skin patterns of females was poorly maintained, and tended to take on a look that was less defined, and with lower contrast. Females shrunk during senescence, and survived four to six weeks after the last eggs were laid. One female did not move for three to four weeks before dying. Senescent males and females typically sat with mantle and head in the den, and the oral surface, beak and suckers exposed (Fig 3C). When in open sand, senescent females sat slightly buried, with the arms intertwined over the head and mantle creating a space presumably used for ventilation.

### Embryos and hatchlings

We lack precise information about the timing of egg maturation. It was not possible to record numbers of eggs laid each day as different groups of eggs were laid next to and among each other. Due to the small number of specimens we did not want to risk damaging the eggs or adults to conduct detailed inspections. We estimate that eyespots began to appear two weeks into development (housed at 20–23°C). Eyespots were yellow-orange in color. At the appearance of eyespots, most embryos were oriented in the capsule in the proximal orientation, with eyespots closest to the stalk. However, a few embryos were situated with the eyes distal to the stalk, which did not appear to be part of the turning process, because it happened so early in development. In the 1970’s hatching began between days 37–38 (housed at 27–28°C). Hatchlings were pelagic ([5]; Fig 5G and 5H).

## Discussion

To our knowledge LPSO exhibits several behaviors not previously described for other octopuses. Mating and egg laying in this species appear to be unique. The beak-to-beak mating posture is similar to the head-to-head mating posture typical in decapod cephalopods (squids and cuttlefishes; [32,33]), especially those that mate directly beak-to-beak (e.g. *Dosidicus gigas* [34]). However, with the exception of LPSO, this mating position appears to be absent among octopuses. All other known octopuses mate using ‘distance’ or ‘mount’ postures, or intermediate positions between the two [32,33,35]. When decapods mate head-to-head the male’s funnel is positioned near the female’s buccal region, where spermatophores are often placed during copulation [33,36,37]. Male squids and cuttlefishes mating in the head-to-head posture can flush the female’s buccal region, clearing the area for placement of his sperm, or potentially removing the existing sperm of rival males [38,39]. Beak-to-beak mating by LPSO would not confer this advantage in sperm-competition to males. Rather than depositing spermatophores for external sperm storage as in decapods, male octopuses place spermatophores internally in the oviducts of the female, which is located inside the mantle and not within reach of male flushing via jetting [33,40]. Unlike with distance and mounting postures observed in other types of octopus, beak-to-beak mating in LPSO frequently involves grappling, and allows rapid full envelopment of the male into the female’s oral web, behaviors that are typically associated with aggressive contests in octopuses [14,27].

We have no direct evidence that the beak-to-beak mating and feeding postures by LPSO confer specific advantages, although we speculate on three non-mutually exclusive hypotheses: allowing for females to maintain their brooding-typical posture while mating and/or eating, allowing for simultaneous feeding by a mating male and female pair, and limitation of mating access to females. The resting posture of brooding LPSO involves the oral surface facing outward while the dorsal mantle faces the eggs. The beak-to-beak posture might facilitate mating with females that are brooding, but still likely to lay more eggs. As with mating in the ‘distance’ position in other octopuses [41], female LPSO can also feed while mating beak-to-beak. This mating position might allow for sucker alignment and simultaneous beak-to-beak feeding associated with mating and/or pairing behavior, including during brooding. The cases of beak-to-beak feeding behavior were observed by a pair that, at other times, mated repeatedly and co-occupied den space. Previously, pair bonding has been noted to occur in this species [5]. However, thus far no observations have been designed to verify this behavioral aspect of LPSO. We do not know if these behaviors are associated with long-term pairings in the wild, as can occur for up to two weeks in group-living *Abdopus aculeatus* [41]. Finally, beak-to-beak mating might allow males to monopolize mating access to females. In octopuses, females can mate with two males simultaneously (one per oviduct), and in some cases more males try to mate with the female than she has oviducts available (*Octopus cyanea* [42]; *Vulcanoctopus hydrothermalis* [43]; *Octopus kaurna* [21], *Octopus bimaculoides* [32]). Beak-to-beak mating might limit access to a mating female to one male at a time because she has one buccal area, a situation that might confer competitive mating advantages in this group-living species.

Egg laying in LPSO is especially prolonged compared to that exhibited by other octopuses. In shallow-water octopuses, female senescence typically begins shortly after egg laying, and death coincides approximately with hatching [31]. This generalization does not apply to LPSO, whose females lay eggs for up to six months and brood for up to eight months. Females continue to feed, lay at least hundreds of additional eggs, and accept additional copulations long after the first eggs have hatched. Although possibly common in deep-sea cirrate octopodids (e.g. *Opistoteuthis* [44,45]), few shallow-water octopuses are known to exhibit extended spawning. Iteroparity, a form of extended spawning in which multiple batches of eggs are laid, is thought to increase lifetime fecundity of very small cephalopods (as in *Octopus chierchiae* [6]), which are limited by ovary space in the mantle cavity [46]. While this tactic would increase long-term fecundity of LPSO as well, this species is of average size rather than small, reaching a mantle length of at least 70 mm while laying eggs. It appears that numerous selective pressures or evolutionary constraints may have led to the rare but phylogenetically widespread expression of this trait.

Although the striking high contrast dark brown and white body color pattern of LPSO is unique among octopuses, similarities in body color pattern and texture can be found. *Octopus chierchiae* exhibits dark bars and stripes, similar to that of LPSO, but over a predominantly pale rather than dark background [6]. *Octopus zonatus*, another Harlequin octopus, bears body patterning similar to *O*. *chierchiae* [2]. The deimatic display of *Abdopus* spp. often involves pale spots over a dark background [47], but resting patterns and skin textures are more complex than those of LPSO [22,47]. Finally *Thaumoctopus mimicus* and *Wunderpus photogenicus* exhibit body patterns that incorporate high-contrast dark brown and pale spots and bars visible at rest [48]. However, in *T*. *mimicus* the elements of these patterns are less clearly delineated [49], and in *W*. *photogenicus* [50] they are reversed, with bars on the arms and pale spots on the mantle rather than vice versa in LPSO. Skin texture of LPSO is not unique. The granular skin, including on the dorso-lateral surfaces of the arms, is similar to that found in *Amphioctopus* spp. [17,51]. LPSO also takes on a resting posture common in *Amphioctopus* (Fig 3C; Figure 5A in [51]; Figure 7 in [52]). However LPSO lacks the flap-like primary papillae in a diamond configuration on the mantle, and dark arm stripes along the dorsolateral edge of arms typically found in *Amphioctopus*. Instead, LPSO bears low conical mantle papillae. Along with *W*. *photogenicus*, *Hapalochlaena lunulata* [53] and *O*. *chierchiae*, LPSO bears individually unique and consistently distinguishable body color patterns. Behavioral and biological similarities with *Octopus chierchiae* are numerous [6] and could possibly be attributed to close phylogenetic relationships (Wright, Caldwell, Ross unpublished data). We have no evidence for speculating on similarities between *T*. *mimicus*, *W*. *photogenicus*, *Amphioctopus* and LPSO other than these octopuses all occupy a sandy bottom habitat [21,51].

On multiple occasions SCUBA divers have found groups of LPSO living in very close proximity to one another ([4,5], collections in 2012–2014), a situation that might have strong implications for behavioral interactions. Many octopuses are known to have an ecologically clumped distribution according to habitat features or resources (e.g. *Abdopus aculeatus*, *Octopus insularis*, *Octopus vulgaris* [22,54,55]). Those populations with grouped dens and/or especially high local densities appear also to exhibit more frequent intraspecific interactions, aggression, and/or more complex mating associations than exhibited by more solitary octopuses [27,41,56–59]. Where suitable habitat is limited to small islands of hard substrate surrounded by a soft substrate, *Octopus tetricus* constructs dens in high densities, which are maintained successively by different individuals [56]. We do not know whether this situation also applies to LPSO. The SCUBA divers who collected LPSO between 2012 and 2014 reported targeting a single aggregation that persisted in the same area for over two years. This observation suggests group site fidelity, which might extend beyond the life span of a single individual. Likewise, we do not know whether LPSO living in groups visually recognize conspecifics, which is associated with reduced physical aggression and increased localized avoidance with familiar individuals in *Octopus vulgaris* [60]. If so, then it is possible that their individually unique body color patterns might facilitate this form of recognition, and allow for the unique suite of interspecific interactions observed here in LPSO, such as repeated mating, and food and den sharing.

## Supporting Information

S1 Movie Larger Pacific Striped Octopus Prey remains carried from den and jetted from area. (MOV)

S2 Movie Larger Pacific Striped Octopus bilateral display with granular texture, while twirling arm tips. Note second individual in neighboring aquarium (upper right of video, not in focus).
(MP4)

S3 Movie Larger Pacific Striped Octopus arm twirling. Visible components include extended eye bar, and granular skin texture.
(MP4)

S4 Movie Larger Pacific Striped Octopus locomotion *slow bounce* posture. (MOV)

S5 Movie Larger Pacific Striped Octopus catching shrimp using arm reach. (MP4)

S6 Movie Larger Pacific Striped Octopus Mating. (MOV)
